# A Novel Approach for Reducing Enteropathogens Using LAB-Fermented Whey Solution and UV-C Light-Activated Gallic Acid

**DOI:** 10.3390/foods15152589

**Published:** 2026-07-23

**Authors:** Luz Alejandra Márquez-Martínez, Xochitl Aparicio-Fernández, Genaro Gustavo Amador-Espejo, Paola Hernández-Carranza, María de Guadalupe Muñoz-Arenas, Irving Israel Ruiz-López, Carlos Enrique Ochoa-Velasco

**Affiliations:** 1 Facultad de Ciencias Químicas, Benemérita Universidad Autónoma de Puebla, Av. San Claudio y 18 Sur Ciudad Universitaria, Puebla 72570, Puebla, Mexico; luz.marquezm@alumno.buap.mx (L.A.M.-M.); paola.hernandezc@correo.buap.mx (P.H.-C.); guadalupe.munoz@correo.buap.mx (M.d.G.M.-A.); 2 Centro Universitario de los Lagos, Universidad de Guadalajara, Av. Enrique Díaz de León No. 1144. Col. Paseos de la Montaña, Lagos de Moreno 47460, Jalisco, Mexico; xochitl.aparicio@academicos.udg.mx; 3 Instituto Politécnico Nacional, Centro de Investigación en Biotecnología Aplicada, Santa Inés Tecuexcomac-Tepetitla, km 1.5, Tepetitla de Lardizabal 90700, Tlaxcala, Mexico; ggamadores@secihti.mx; 4 Facultad de Ingeniería Química, Benemérita Universidad Autónoma de Puebla, Av. San Claudio y 18 Sur Ciudad Universitaria, Puebla 72570, Puebla, Mexico

**Keywords:** *Lactobacillus*, probiotics, natural antimicrobial compounds, in vitro digestion model

## Abstract

This study evaluates the combined antibacterial activity (AA) of lactic acid bacteria (LAB) and UV-C light-activated gallic acid (AGA) against two common enteropathogens (*Escherichia coli* and *S.* Typhimurium) in terms of microbial growth and survival during gastrointestinal simulation (GS). The LAB were selected based on their capacity to ferment soy and whey concentrates in monoculture and co-culture. The resulting LAB-fermented medium (LABF) was evaluated for its antibacterial properties against enteropathogens and combined with AGA to determine its effects during the GS. Results indicated that the whey solution supported the highest growth of LAB co-culture (>10^7^ CFU/mL). LABF showed AA in a dose-dependent manner, achieving >2-log reductions. Although AGA exhibited strong AA, when combined with LABF, its antimicrobial activity under GS was null, with a complete microbial recovery after 24 h of GS. Therefore, the application of LABF plus AGA in the in vitro gastrointestinal system needs further study to avoid loss of antibacterial activity during gastric transit.

## 1. Introduction

Bacterial infections and antibiotic resistance are a global health concern, linked to over 1 million deaths in 2021 [1]. According to the World Health Organization (WHO), multidrug-resistant pathogens cause more than 700,000 deaths annually, and projections indicate this number could reach 10 million by 2050 if action is not taken [2]. Specifically, the US Centers for Disease Control and Prevention (CDC) reports that foodborne pathogens and their toxins cause about 48 million hospitalizations and approximately 3000 deaths each year [3]. This problem is primarily attributed to the widespread use of antibiotics in both human medicine and agriculture, as well as to the rapid adaptability of microorganisms to novel antibiotics. Mechanisms of bacterial resistance include genetic mutations, efflux pumps, enzymatic activation, target modifications, and biofilm production [4,5]. Additionally, outer membrane vesicles may facilitate genetic exchange, disseminating resistance traits among bacterial populations [2].

The food industry has explored a range of treatments to reduce foodborne illnesses and food spoilage. Both novel and conventional technologies, as well as emerging antimicrobials, have been extensively studied [3,6]. In this last aspect, the use of natural antimicrobials or biopreservatives derived from microorganisms, plants, and their derivatives has gained importance as a promising alternative to conventional therapies due to their ability to target multiple pathways simultaneously. The most significant mechanisms of inhibition include cell wall damage, disruption of protein synthesis, and interference with biofilm production [7].

Probiotics are living microorganisms that have been shown to improve human health when consumed regularly in sufficient quantities (>10^9^ CFU/mL or g) [8]. Documented benefits of probiotics include reducing the incidence of gastrointestinal disorders, lowering cholesterol levels, and mitigating the risks associated with type 2 diabetes and obesity. Additional effects include prevention of urinary tract infections, enhancement of dental health and immune system function, and antipathogenic, antiallergic, anti-inflammatory, anticancer, and angiogenic activities [9,10]. Probiotics also exhibit antimicrobial activity in the human body and as biopreservatives in foods. In humans, competing with pathogens for nutrients and for receptor-binding sites in the gut, metabolite productions such as organic acids, peptides, amphiphilic membrane-active biosurfactants, and hydroperoxides are the most relevant antimicrobial factors [10], whereas, in food products, the antimicrobial effect of probiotics has been focused on the metabolite’s application (cell-free supernatants), where organic acids (lactic, acetic, propionic acids) and bacteriocins (nisins, lactacins, enterocins, colicins, etc.) are the most common [10,11]. In this sense, the inhibitory action of organic acids is based on the reduction in pH and ionization of cell membranes, affecting cell metabolism and permitting the introduction of oxidized compounds, while bacteriocins act by forming pores in the cell wall or blocking protein and DNA biosynthesis [12]. Among probiotics, lactic acid bacteria, including *Lactobacillus*, *Lactococcus*, *Pediococcus*, *Weissella*, and *Leuconostoc*, are the most used as biopreservatives against foodborne pathogens and food spoilage microorganisms [12,13,14].

Gallic acid (3,4,5-trihydroxybenzoic acid) is a phenolic compound widely distributed in fruits, plants, and their derivatives [15]. It is well recognized for its potent biological activity and therapeutic versatility, with its antioxidant and anti-inflammatory properties among the most researched [16]. In this sense, its consumption has been associated with reducing intestinal bowel diseases by inhibiting key signaling pathways and producing anti-inflammatory molecules [17,18,19]. Gallic acid also exhibits antimicrobial activity, which can be potentiated using ultraviolet light [20]. In this aspect, some studies have indicated that UV-A and UV-C increase the antimicrobial activity of gallic acid in in vitro assays [20,21,22,23] and to disinfect plants such as tomato [24] and lettuce leaves [25]. Gallic acid has also been used to improve the antimicrobial activity of several synthetic and natural antimicrobials. For example, the combination of gallic acid with ceftiofur sodium and tetracycline enhances antimicrobial activity against *Escherichia coli* [26]. The combination of gallic acid with azithromycin enhances antimicrobial activity against *Staphylococcus aureus* and *Pseudomonas aeruginosa* [27], while the use of gallic acid plus natural antimicrobials such as carvacrol enhances the antimicrobial activity against *P. aeruginosa* and *S. aureus* [28], with thymol against *E. coli* O157:H7 and *S. aureus* in fresh-cut tomatoes [29], and with gadolinium against resistant *Salmonella* [30].

The combination of gallic acid with probiotics or their fractions has not been explored, which can be of utmost importance since gallic acid has also shown gastrointestinal benefits such as protecting the mucosal layer of the gastrointestinal tract, reducing oxidative stress, lipid peroxidation, and the inflammatory process [31]. In addition, according to Andrade et al. [32], co-fermentation of apple–cashew with probiotics enhances the bioaccessibility of gallic acid. Therefore, this study aimed to evaluate the antimicrobial activity of UV-C light-activated gallic acid, in combination with a probiotic culture, against two common enteropathogens (*E. coli* and *Salmonella* Typhimurium) in terms of microbial growth and in a gastrointestinal simulation. To attain this goal, the following steps were taken: (i) select the probiotic, initial inoculum, and growth medium and concentration to use in the fermentation process; (ii) evaluate the antibacterial activity of the selected LAB-fermented medium and its compounds, biomass and cell-free supernatant, against two enteropathogens (*E. coli* and *S.* Typhimurium); and (iii) evaluate the combination of UV-C light-activated gallic acid as a basis for LAB-fermented medium against two enteropathogens under gastrointestinal simulation.

## 2. Materials and Methods

### 2.1. Overview of the Study

**Figure 1:**
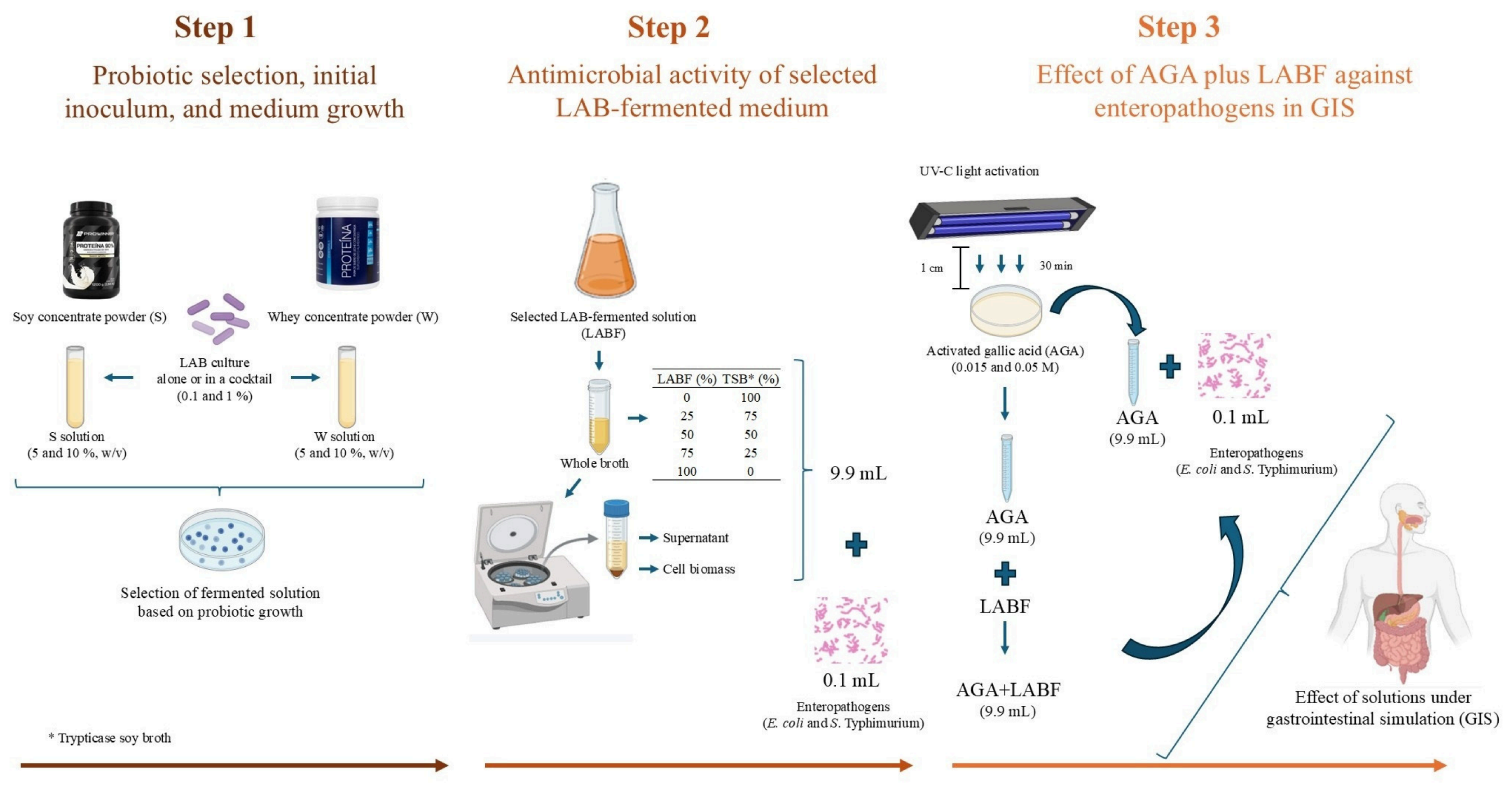
Schematic representation of this research.

This study was conducted in three steps (Figure 1): (1) selection of LAB in mono- or co-culture and initial inoculum quantity, as well as the growth medium and concentration that present the highest probiotic growth during the fermentation process. (2) Evaluation of the antibacterial activity of selected LAB-fermented (LABF) medium and its compounds, cell-free supernatant, and biomass, against two enteropathogens (*Escherichia coli* and *Salmonella* Typhimurium). (3) Evaluation of gastrointestinal simulation on the microbial population of LABF, *E. coli*, and *S.* Typhimurium, either alone or combined with UV-C light-activated gallic acid (AGA).

### 2.2. Materials and Reagents

Whey and soy protein concentrates (80–85% protein) were obtained from New Life Suplementos (Monterrey, Mexico) and Prowinner (Mexico City, Mexico), respectively. Gallic acid was obtained from Sigma-Aldrich (Burlington, MA, USA). Trypticase soy broth, MRS broth, MRS agar, eosin methylene blue (EMB) agar, and Salmonella–Shigella agar were obtained from BD Bioxon (Col. Lomas de Chapultepec, Mexico City, Mexico).

### 2.3. Microorganisms

Probiotics [LAB, *Lactobacillus acidophilus* (NRRL B-4495), *Lacticaseibacillus casei* (NRRL B-1922), *Limosilactobacillus reuteri* (NRRL B-14171), *Lactiplantibacillus plantarum* (NRRL B-4496), and *Lacticaseibacillus rhamnosus* (NRRL B-442)] and enteropathogens (*Escherichia coli*, ATCC 25922, and *Salmonella* enterica serovar Typhimurium, ATCC 14028) were obtained from the microbial collection of the Chemistry Science Faculty of the Benemerita Universidad Autonoma de Puebla (Puebla City, Mexico). Microorganisms [1 mL of cell suspension plus 1 mL of aqueous glycerol (20% *v*/*v*)] in the early stationary phase were maintained in Eppendorf tubes at −18 °C. For reactivation, probiotic strains were incubated in MRS broth. At the same time, enteropathogens were cultured in trypticase soy broth at 37 ± 2 °C for 24 h using an incubator model INO650M (PRENDO, Puebla, Mexico). After the culture time, the microbial loads (1.0–2.0 × 10^9^ CFU/mL) of both probiotics and enteropathogens were determined by diluting in peptone water to obtain an appropriate count (30–300 CFU/mL). Samples were plated in MRS agar for probiotics, EMB agar for *E. coli*, and Salmonella–Shigella agar for *S.* Typhimurium and counted after 24 h of culture at 37 ± 2 °C. Reactivated microorganisms were immediately used for antibacterial tests.

### 2.4. Evaluation of Probiotic Mono- and Co-Cultures at Different Inoculum Amounts and Fermentation Media

Probiotics mono- and co-cultures were evaluated in two different media at different concentrations. In brief, 100 mL of whey and soy concentrates at 5 and 10% (*w*/*v*) was mono-cultured (1.0 mL of probiotic) and co-cultured (0.2 mL of each probiotic). The solutions were incubated at 37 ± 2 °C for 24 h, then diluted in peptone water to obtain an appropriate count (30–300 CFU/mL). Samples were plated in MRS agar and incubated under anaerobic conditions. The probiotics were counted, and the results were expressed as log CFU/mL. Once the culture conditions (mono- or co-culture, media, and concentration) that yielded the highest LAB growth were selected, the initial inoculum size was evaluated. Briefly, 0.1 or 1.0% (*v*/*v*) of probiotics was added to 100 mL of the selected medium, and the solutions were subsequently treated as described above. For subsequent assays, the initial probiotic inoculum (mono- or co-cultured) and the culture medium and concentration that supported the highest probiotic growth were selected. This fermentation condition was designed as a lactic acid bacteria-fermented medium (LABF). The fermentation process on the selected media was measured by pH using a pH meter, and the titratable acidity was determined by titration with 0.1 N NaOH solution, reported as lactic acid (%, *v*/*v*).

### 2.5. Effect of LABF on the Microbial Growth of Enteropathogens

The antibacterial activity of the LABF against enteropathogens was evaluated. Tubes containing 9.9 mL of 24 h LABF diluted in trypticase soy broth to final concentrations of 0, 25, 50, 75, and 100% were inoculated with 0.1 mL of *E. coli* or *S.* Typhimurium suspension (1.0–2.0 × 10^9^ CFU/mL). To evaluate the effects of LABF components (supernatant and biomass), a centrifugation process was performed. The LABF was centrifuged at 1968 *g* for 10 min using a centrifuge model XC-2450 (Premiere, Texas City, TX, USA). The cell-free supernatant fraction (PLABF) and the precipitate (cell biomass, CLABF) were processed as follows. For the PLABF, 9.9 mL of supernatant was individually inoculated with 0.1 mL of the enteropathogens suspension. For the CLABF, the precipitate was resuspended in sterile distilled water to a final volume of 9.9 mL and then individually inoculated in the same manner. In addition, tubes containing 9.9 mL of LABF were sterilized (121 °C for 15 min) and inoculated with 100 µL of enteropathogens to evaluate the antibacterial activity of inactivated cells (SLABF). All tubes were incubated for 24 h at 37 ± 2 °C. Then, 1 mL of each sample was diluted in peptone water to achieve an appropriate count (30–300 CFU/mL). Samples were plated on EMB or Salmonella–Shigella agar for *E. coli* and *S.* Typhimurium, respectively. Petri dishes were incubated at 37 ± 2 °C for 24 h, and results were expressed as the logarithmic reduction in colony-forming units per milliliter (CFU/mL).

### 2.6. Effect of Gallic Acid and Its Combination with LABF on the Microbial Growth of Enteropathogens

To evaluate the effect of gallic acid alone and when used for the formulation of LABF against enteropathogens, several experiments were conducted (Table 1). Aqueous gallic acid solutions at two concentrations (0.015 and 0.05 M) were UV-C light-activated following the methodology proposed by Luna-Domínguez et al. [20]. The selected medium was mixed using activated gallic acid (AGA) and nonactivated gallic acid (NAGA) solutions as solvents. Then, the probiotic was inoculated, and its growth was evaluated as previously described. The combined effect of gallic acid and LABF against two enteropathogens (*E. coli* and *S.* Typhimurium) was evaluated using the methodology described in the previous section. AGA with the highest microbial inactivation was selected for further assays. NAGA and non-fermented medium (NFM) were used as controls.

### 2.7. Effect of Gastrointestinal Simulation on the Survival of Probiotics and Enteropathogens

The effect of gastrointestinal simulation on probiotics (LABF) and enteropathogens was evaluated according to the standardized static in vitro digestion methodology proposed by He et al. [33]. In brief, the gastric solution was formulated by diluting 3.2 g of pepsin and 2 g of NaCl in 1 L of sterile distilled water at pH = 2 (adjusted with HCl). The intestinal solution was formulated by dissolving 10 g of pancreatin and 6.8 g of K_2_HPO_4_ in 1 L of sterile distilled water (pH = 7.0, adjusted with NaOH). One mL of the LABF, *E. coli*, and *S.* Typhimurium and their combination LABF (0.5 mL) plus *E. coli* (0.5 mL) and LABF (0.5 mL) plus *S.* Typhimurium (0.5 mL) at the early stationary phase (24 h cultured) was mixed with 9 mL of gastric solution for 2 h (37 ± 2 °C at 110 rpm). Then, 1 mL of this mixture was combined with 9 mL of intestinal solution and incubated at 37 ± 2 °C (110 rpm) for 24 h. Samples were taken after 2 h of gastric simulation and after 3 h and 22 h of intestinal simulation. The samples were plated in MRS, EMB, or Salmonella–Shigella agar, depending on the type of microorganism: probiotics, *E. coli*, or *S.* Typhimurium, respectively. Petri dishes were incubated at 37 ± 2 °C for 24 h (anaerobic conditions for probiotics), and results were expressed as log (CFU/mL).

### 2.8. Effect of Gastrointestinal Simulation on Enteropathogens Treated with AGA Alone or Combined with LABF

The effect of selected AGA alone, as well as its use for LABF growth, on the survival of enteropathogens during gastrointestinal simulation was evaluated. To conduct this analysis, three systems were developed and added with enteropathogens: (1) AGA solution, (2) AGA solution plus NFM, and (3) AGA solution as the basis for LABF (incubated for 24 h at 37 ± 2 °C). Then, both enteropathogens were separately inoculated into the three previous solutions and submitted to the gastrointestinal process. Probiotics, *E. coli*, and *S.* Typhimurium were plated in MRS, EMB, and Salmonella–Shigella agar, respectively. Petri dishes were incubated at 37 ± 2 °C for 24 h, and the results were expressed as log (CFU/mL).

### 2.9. Statistical Analysis

The experiments were performed in triplicate, and all assays were conducted in duplicate. The results were analyzed using one-way analysis of variance (ANOVA) with Tukey’s test for pairwise comparisons in Minitab v.15 (Minitab Inc., State College, PA, USA).

## 3. Results and Discussion

### 3.1. Effect of Fermentation Media and Inoculum Quantity on Probiotic Growth in Monoculture and Co-Culture

**Figure 2:**
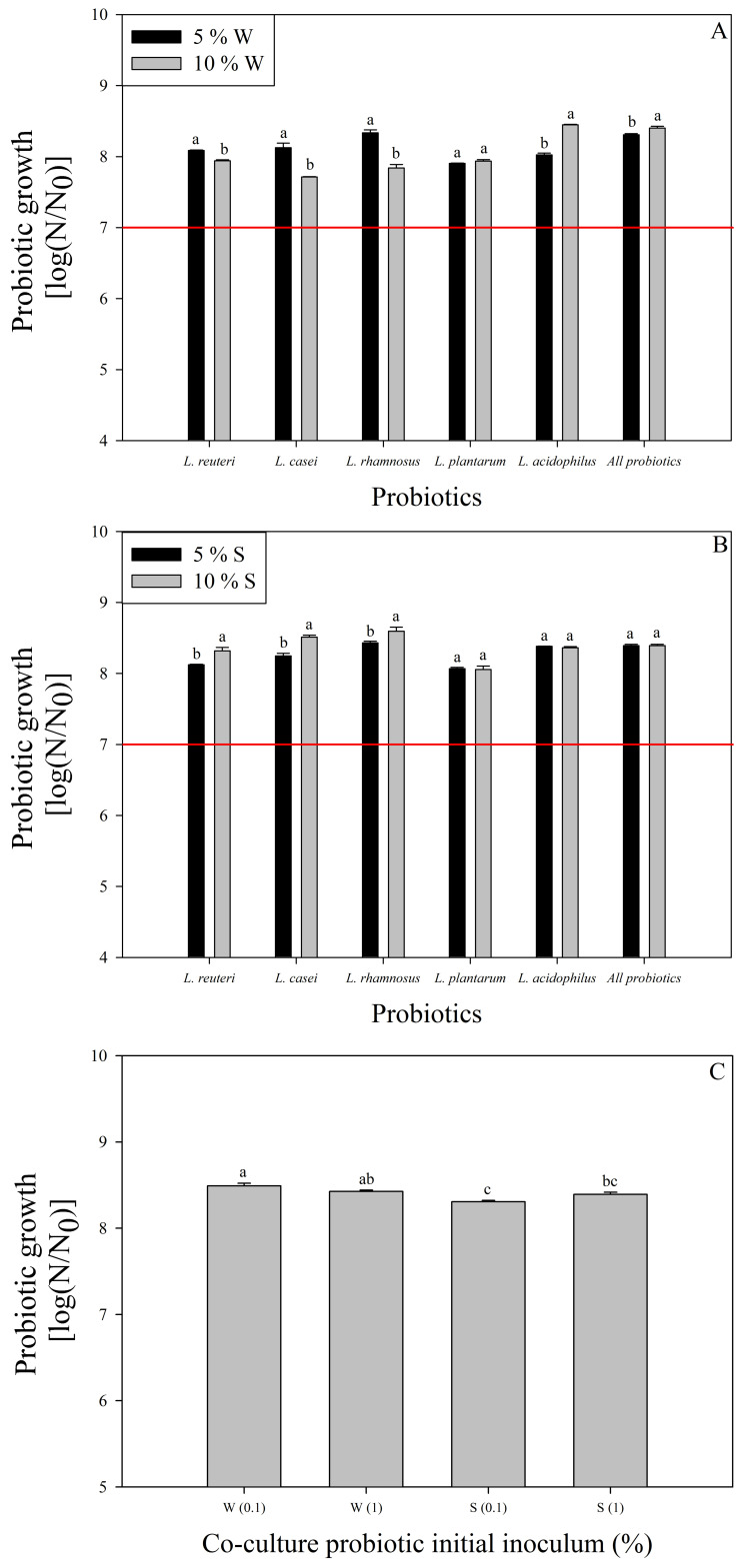
Probiotic growth using (**A**) whey concentrate (W, 5 and 10%), (**B**) soy concentrate (S, 5 and 10%), and (**C**) initial inoculum (0.1 and 1%) at 5% of whey and soy concentrates. Bars indicate standard deviation. Different letters (a, b, or c) among microorganisms or fermented media indicate statistically significant differences (*p* < 0.05). The red line indicates the minimum count to be considered a food with high probiotic content (>10^7^ CFU/g).

Figure 2 shows the effect of fermentation media and concentration, as well as the initial inoculum, on the growth of probiotics in monoculture and co-culture. As noted, in all media, the probiotic count was higher than 7 logs (1 × 10^7^ CFU/mL), indicating that the evaluated media are adequate for probiotic growth, considering both media as products with a high probiotic count according to Hill et al. [34]. They categorized fermented foods into three different groups. Food with low (<10^4^ CFU/g), medium (10^4^–10^7^ CFU/g), and high (>10^7^ CFU/g) probiotic count. On the other hand, increasing the amount of whey concentrates used in the solution formulation increased the probiotic count when used in LAB monocultures (*p* < 0.05). However, the probiotic co-culture maintained the count regardless of the whey amount. This phenomenon can be attributed to the combination of both homo- and heterofermentative LAB, which improve microbial growth due to mutualism, an interaction where the metabolites released (amino acids, vitamins, growth factors, and carbon and nitrogen sources) can be used by each other to meet their own nutrient requirements [35]. This finding was also noted when soy concentrate was fermented in co-culture (Figure 2B). It is interesting to note that increasing the amount of concentrated soy in fermented media significantly affects the probiotic growth in most LAB. This may be due to the solubility and water-holding capacity of proteins present in soy concentrate [36]. Thus, increasing the soy concentrate amount may reduce protein solubility, increase water-holding capacity, and reduce the use of nutrients for some LAB. In this respect, the probiotic co-culture reached a higher count due to the explanation provided before, and *L. acidophilus* may show a higher probiotic count due to its slow growth rate, which may reduce the nutrient needs at a given time during the fermentation process [37].

Figure 2C shows the probiotic co-culture growth inoculated at two concentrations (0.1 and 1%) on whey and soy concentrate at 5%. As observed, when probiotics were inoculated in co-culture, the microbial population was higher than 8 logs, regardless of the initial inoculum and fermented media used. However, the higher probiotic count was observed with whey concentrate, which can be attributed to the residual content of lactose, the main source of nutrients for lactic acid bacteria. In this sense, Garrote Achou et al. [38] indicated that both carbon and nitrogen sources are important not only for obtaining higher probiotic growth but also for affecting the antimicrobial activity response. Based on these results, a co-culture (1%) of lactic acid bacteria inoculated in 5% whey concentration (LABF) was selected for further analysis.

### 3.2. Antibacterial Activity of LABF Against Enteropathogens

**Figure 3:**
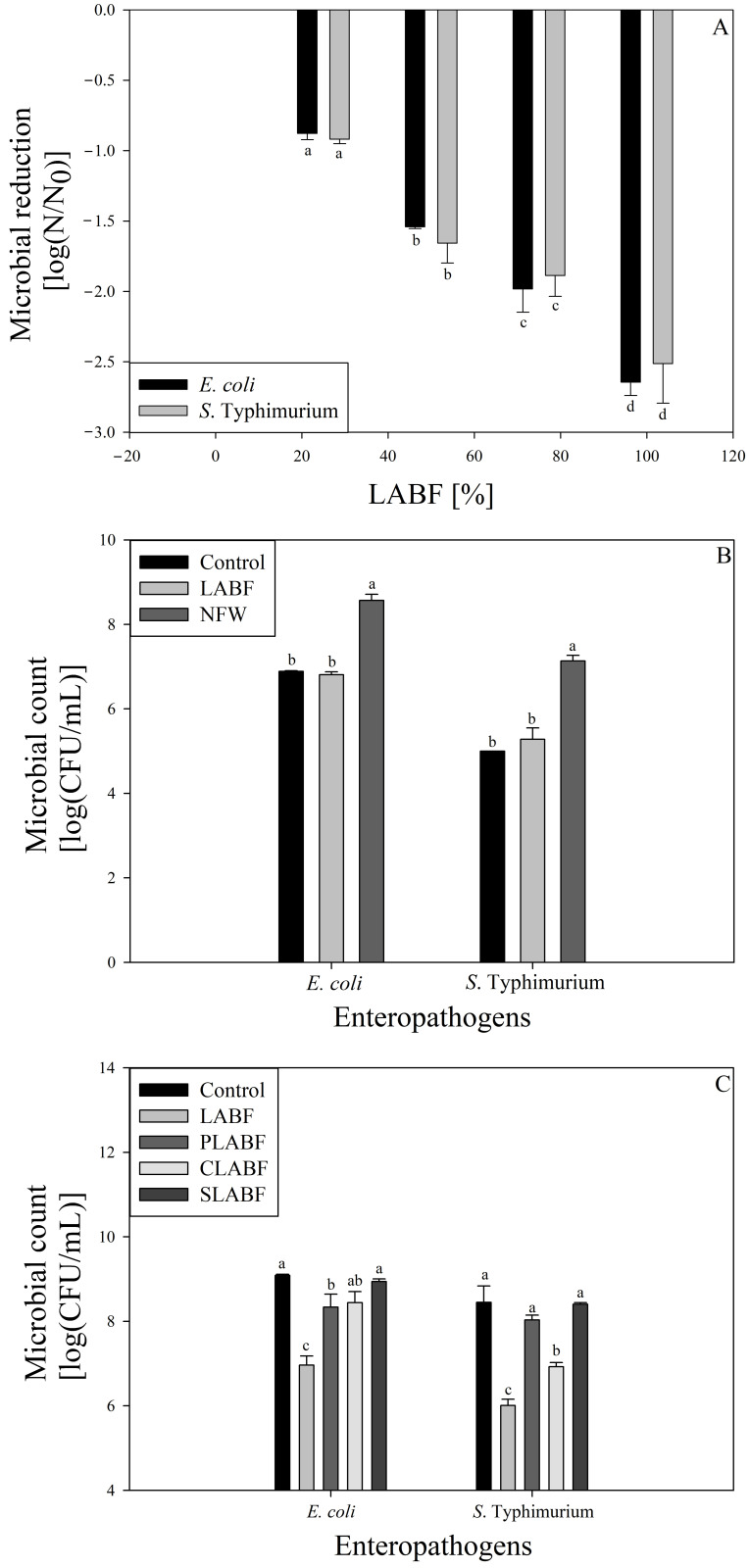
Effect of LABF and its components on the microbial inactivation of enteropathogens. (**A**) Effect of concentration of 24 h LABF against *E. coli* and *S.* Typhimurium. (**B**) A 24 h co-culture of LABF with *E. coli* or *S.* Typhimurium. Trypticase soy broth and NFW were used as controls. (**C**) A 24 h co-culture of LABF, supernatant LABF (PLABF), precipitate LABF (CLABF), and sterilized LABF (SLABF). Trypticase soy broth was used as a control. Bars indicate standard deviation. Different letters (a–d) indicate significant statistical differences (*p* < 0.05) between microorganisms.

The antibacterial activity of the chosen medium (whey concentrate, 5% *w*/*v*) co-cultured with probiotics was evaluated against *E. coli* and *S.* Typhimurium. The effect of LABF on the microbial inactivation of both microorganisms is shown in Figure 3A. As observed, increasing the LABF concentration increases microbial reduction, reaching maximum decreases of 2.32 and 2.71 logs for *E. coli* and *S.* Typhimurium, respectively. The antibacterial activity of probiotics against pathogenic microorganisms has been widely studied, and it is conferred through several mechanisms. Among them, nutrient competition, the activation of silent genes, and metabolites produced by probiotics, such as bacteriocins, exopolysaccharides, enzymes, and organic acids, including short-chain fatty acids, which can present not only antibacterial activity but also a change in the medium characteristics, for example, acidity, may be the most important factors for antibacterial activity [30,39]. However, given that the LAB-fermented medium had a pH = 4.52 ± 0.02 and a titratable acidity of 0.18 ± 0.01% (lactic acid) and considering that both microorganisms can grow under these conditions [40,41], the increase in the titratable acidity and the reduction in pH during the fermentation process cannot be considered to be the antimicrobial factor of the LAB-fermented solution.

The inoculation of *E. coli* or *S.* Typhimurium into the LABF was evaluated to determine growth interactions between enteropathogens and probiotics (Figure 3B). The results indicated that enteropathogens can grow in the control solution using whey nutrients. However, when the LABF was inoculated as a co-culture with enteropathogens, no growth was detected (*p* < 0.05), indicating that metabolites produced by the probiotic cocktail during the fermentation inhibit the enteropathogens’ growth. As mentioned before, during the culture of probiotics, several metabolites are produced, including biosurfactants, peptides, and H_2_O_2_, among others. It is interesting to note that the antibacterial activity of probiotics may depend on the growth phase used and, consequently, the metabolites produced. This indicates that a bacteriostatic effect is observed when microorganisms are co-cultured (Figure 3B). In contrast, a bactericidal effect is observed when probiotic metabolites are previously produced and then used as antibacterial [42]. Moreover, it is important to highlight that the bactericidal effect obtained in this study may be due to the use of a consortium. In this respect, according to Fijan et al. [11], the use of multi-strain probiotics exhibited greater antimicrobial activity than single-strain probiotics.

To determine the contribution of cell-free supernatants and cell biomass to the inactivation of both enteropathogens, the LABF was separated into two fractions (Figure 3C): the supernatant (PLABF) and the precipitate (CLABF). In addition, the LABF was thermally treated to determine the antibacterial effect of inactivated cells (SLABF). As observed, microbial inactivation depends on the fermented fraction and the heating process. In this sense, thermally treated LAB-fermented solution (SLABF) did not exhibit antibacterial activity against either of the two microorganisms, which reinforces the antimicrobial activity associated with the metabolites produced during the fermentation process, because pH (4.50 ± 0.02) and titratable acidity (0.18 ± 0.00%, lactic acid) did not change due to the thermal treatment (*p* > 0.05). However, cell-free supernatant and cell biomass significantly affected (*p* < 0.05) *E. coli* and *S.* Typhimurium, respectively. Tong et al. [43] pointed out that novel postbiotics from *Bacillus amyloliquefaciens* and *L. plantarum* SN4 showed strong antibacterial activity against *E. coli* compared to *S.* Typhimurium. They noted that postbiotics disrupted the outer cell membrane, making it rough and broken, and leading to the leakage of cell contents. In addition, *E. coli* showed higher DNA damage than *S.* Typhimurium. On the other hand, the antibacterial activity of centrifuged cell biomass may be due to nutrient competition. In this respect, according to Do et al. [44], several strains of *E. coli*, including probiotic strains, contain more iron siderophores and sugar transporters than *Salmonella*, which may limit their growth under nutrient competition [45]. It is interesting to note that the higher microbial inactivation was observed when LABF was used whole against both microorganisms, which is attributed to the combination of antimicrobial effects described above.

### 3.3. Effect of Gastrointestinal Simulation on Probiotics, E. coli, and S. Typhimurium at Different Digestion Times

**Figure 4:**
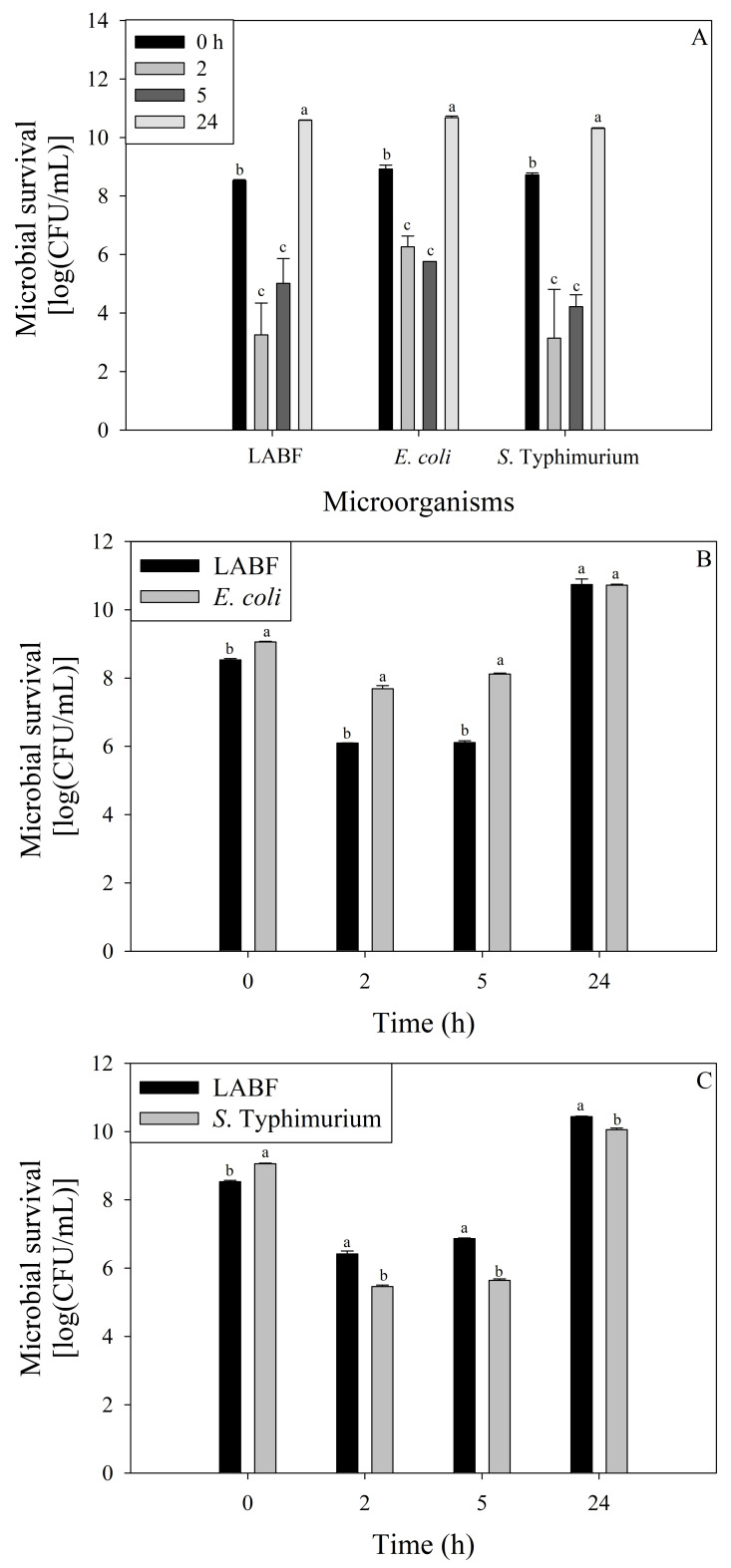
Effect of gastrointestinal simulation on microbial survival inoculated as (**A**) monoculture (LABF, *E. coli*, and *S.* Typhimurium), (**B**) LABF plus *E. coli*, and (**C**) LABF plus *S.* Typhimurium during 24 h. Bars indicate standard deviation. Different letters (a, b, and c) indicate significant statistical differences (*p* < 0.05) between microorganisms.

Figure 4 shows the effect of static in vitro gastrointestinal simulation on the probiotics and enteropathogens at different digestion times, either in monoculture (Figure 4A) or in co-culture (Figure 4B,C). As observed, regardless of the kind of microorganism, the effects of gastrointestinal fluids are similar. This means that during the first two hours of gastrointestinal simulation (gastric phase), a significant reduction in microbial count was detected due to the pH of the gastric solution (*p* < 0.05). This inactivation reduced the microbial count to 3.25 ± 1.09, 6.26 ± 0.37, and 3.14 ± 1.66 logs for probiotics, *E. coli*, and *S.* Typhimurium, respectively. As observed, *E. coli* showed the highest resistance to the acidic condition of the gastric solution, attributed to its higher acid resistance at different pH levels compared to other microorganisms, such as *Salmonella aberdeen* [46]. This trend is also noted when enteropathogens were co-cultured with the LABF. This means that *E. coli* presented higher resistance, followed by probiotics, and finally, *S.* Typhimurium exhibited the lowest pH resistance. However, after 24 h of gastrointestinal simulation, the microbial load of all microorganisms recovered even more than that inoculated at the beginning of the simulated gastrointestinal process. The results obtained were different from those reported by other authors, such as those observed by Tian et al. [47]. They reported that *E. coli* CVCC 1382 and *Salmonella pullorum* CVCC 525 were significantly reduced during co-culturing with probiotics. However, the co-culture process was conducted using MRS broth, a medium used for probiotic culture, while in this study, microorganisms were placed under simulated gastrointestinal conditions. Interestingly, we observed that microorganisms can recover in the intestinal fluid. This phenomenon is reported in various studies on probiotics submitted to in vitro gastrointestinal simulation, attributed to the microorganism’s ability to grow after stress induced by gastric fluid [48].

### 3.4. Evaluation of the Effect of Gallic Acid on the Probiotics’ and Enteropathogens’ Survival During the Gastrointestinal Simulation

Table 2 presents the effects of various treatments on the growth/inactivation of enteropathogens. As observed, in the nonfermented medium (S1, NFM), enteropathogens grew to attain microbial populations higher than 7 logs (CFU/mL), while in LABF (S2), microbial inhibition was detected, resulting in more than 2 and 3 log reductions for *E. coli* and *S.* Typhimurium, respectively. On the other hand, the use of gallic acid (S3) significantly reduced the enteropathogen counts, showing microbial populations in the range of 1.85 and 0.15 logs in *E. coli* at 0.015 and 0.05 M, respectively, while a higher inactivation was observed in *S.* Typhimurium, showing values of 0.54 for the lowest gallic acid concentration, while at 0.05 M, no microbial growth was observed. The irradiation process significantly improves the antibacterial properties of gallic acid, presenting complete inactivation of both pathogens, regardless of the gallic acid concentration (S4). In this sense, some authors have pointed out that the UV-C light activation process significantly improves the antibacterial properties of gallic acid due to the reactive oxygen molecules produced, which can inactivate microbial cells through a superoxidation process [20,21].

On the other hand, when treatments were combined (S5 to S8), the use of the highest concentration of gallic acid significantly improved the antibacterial properties of LABF (S2 versus S7 and S8) against both microorganisms, showing microbial reduction higher than 5 logs (CFU/mL). However, the inactivation observed was lower than that attained when gallic acid (0.05) was used alone (S3 and S4) regardless of the UV-C light activation process. This phenomenon may be attributed to the binding (hydrogen bonding and hydrophobic interactions) of gallic acid to the proteins present in the LABF. In this sense, since the phenolic hydroxyl group of gallic acid is bound to the hydrophobic region of whey protein, the availability of hydroxyl groups to act as toxic agents for microorganisms and/or to produce reactive oxygen species is reduced, which consequently reduces its antimicrobial activity [49,50].

**Figure 5:**
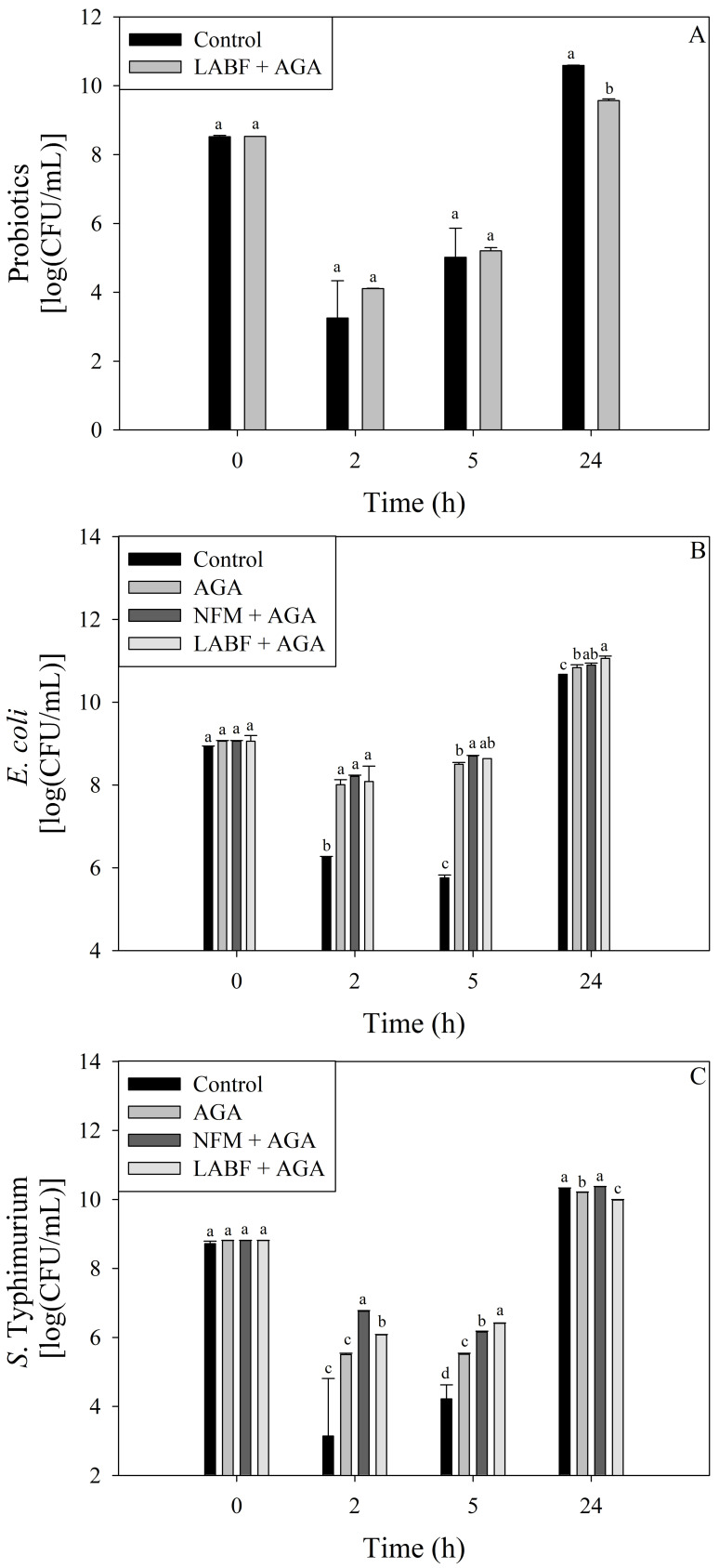
Effect of gastrointestinal simulation on (**A**) probiotics, (**B**) *E. coli*, and (**C**) *S.* Typhimurium treated with activated gallic acid (AGA), LAB-fermented (LABF) plus AGA, and non-fermented medium (NFM) plus AGA. Bars indicate standard deviation. Different letters (a–d) indicate significant statistical differences (*p* < 0.05) between media.

Based on these results, the evaluation of selected solutions against enteropathogens under gastrointestinal simulation was assessed. Nevertheless, an inhibition of the antibacterial properties of activated AGA and LABF plus AGA was noted (Figure 5B,C), showing that the pH of the gastric solution significantly decreased the enteropathogens population compared to the use of AGA and LABF. The results may be explained because both the AGA solution and LABF have a higher pH than the formulated gastric solution (pH = 2.0), reducing the natural antibacterial activity of acidity in this solution. In addition, although the interaction of LABF with AGA may affect the isoelectric point of whey, it is known that reducing the pH of a protein solution can decrease the protein solubility, which can also affect the antimicrobial activity of the LABF plus AGA solution [51].

## 4. Conclusions

The results obtained in this study indicate that the whey solutions showed the highest probiotic growth compared to soy concentrate, and co-culture inoculation showed higher growth than inoculation alone. The LABF exhibited dose-dependent antibacterial activity against both enteropathogens, with a greater effect against *S.* Typhimurium than *E. coli*. On the other hand, AGA solution displayed complete microbial inactivation when used at the highest concentration tested (0.05 N). However, when it was combined with LABF, its antibacterial activity decreased. Unfortunately, the antibacterial activity of both AGA and LABF, whether alone or combined, was inhibited by the gastric fluid from the in vitro gastrointestinal simulation, preventing the natural inhibition associated with this metabolic step. Therefore, to avoid antibacterial inactivation during the simulated gastrointestinal system, further studies are needed.

## Figures and Tables

**Table 1:** Antimicrobial activity of gallic acid (GA) alone and combined with LAB-fermented medium (LABF) against enteropathogens in gastrointestinal simulation.

Treatment	NFM	LABF	GA	AGA
S1	X			
S2		X		
S3			X	
S4				X
S5	X		X	
S6	X			X
S7		X	X	
S8		X		X

NFM: Non-fermented medium. LABF: LAB-fermented medium. GA: Gallic acid (0.015 and 0.05 M). AGA: Activated gallic acid (0.015 and 0.05 M).

**Table 2:** Effect of LAB-fermented medium and gallic acid against *E. coli* and *S.* Typhimurium *.

Systems	*E. coli* [log (CFU/mL)]		*S.* Typhimurium [log (CFU/mL)]	
	0.015 M	0.05 M	0.015 M	0.05 M
S1	8.38 ± 0.07 a	8.38 ± 0.07 a	7.40 ± 0.00 a	7.40 ± 0.01 a
S2	6.23 ± 0.02 b	6.23 ± 0.05 b	4.38 ± 0.06 c	4.38 ± 0.06 b
S3	1.85 ± 0.08 c	0.15 ± 0.21 d	0.54 ± 0.09 d	NG
S4	NG	NG	NG	NG
S5	6.36 ± 0.03 b	2.32 ± 0.03 c	7.15 ± 0.04 ab	3.35 ± 0.01 c
S6	6.26 ± 0.02 b	2.39 ± 0.02 c	6.81 ± 0.05 b	2.54 ± 0.09 d
S7	6.04 ± 0.02 b	2.09 ± 0.02 c	4.20 ± 0.01 c	2.48 ± 0.33 d
S8	6.10 ± 0.04 b	1.45 ± 0.21 c	4.11 ± 0.01 c	2.16 ± 0.18 d

* Average ± standard deviation. Different letters in the same column indicate significant statistical differences (*p* < 0.05). NG: No growth.

## Data Availability

The original contributions presented in this study are included in the article. Further inquiries can be directed to the corresponding authors.

## Abbreviations

The following abbreviations are used in this manuscript:

AA	Antibacterial Activity
LAB	Lactic Acid Bacteria
LABF	Lactic Acid Bacteria-fermented Medium
PLABF	Cell-free supernatant
CLABF	Cell-biomass
SLABF	Inactivated cells
NFM	Non-fermented Medium
GA	Gallic Acid
AGA	Activated Gallic Acid
NAGA	Nonactivated Gallic Acid
GS	Gastrointestinal Simulation
